# Supplementary material for: Targeted Delivery of Butyrate Improves Glucose Homeostasis, Reduces Hepatic Lipid Accumulation and Inflammation in db/db Mice
Source: Int J Mol Sci. 2023 Feb 25;24(5):4533. doi: 10.3390/ijms24054533 (PMC10002599; doi:10.3390/ijms24054533)
Supplement: Supplementary file 1 [file ijms-24-04533-s001.zip › ijms-2219140-supplementary.pdf]

Targeted delivery of butyrate improves glucose homeostasis, reduces hepatic lipid accumulation and inflammation in db/db mice

Signe Schultz Pedersen, Michala Prause, Christina Sørensen, Joachim Størling, Thomas Moritz, Eliana Mariño and Nils Billestrup

Supplementary Materials

List of materials included:

<u>Figures:</u>

Figure S1. Baseline characteristics of db/db mice.
Figure S2. Cytokines in plasma.

<u>Tables:</u>

Table S1. Diet formulations.
Table S2. Primers used for RT-qPCR on liver and fat tissue.
Table S3. Primers used for RT-qPCR on islets.

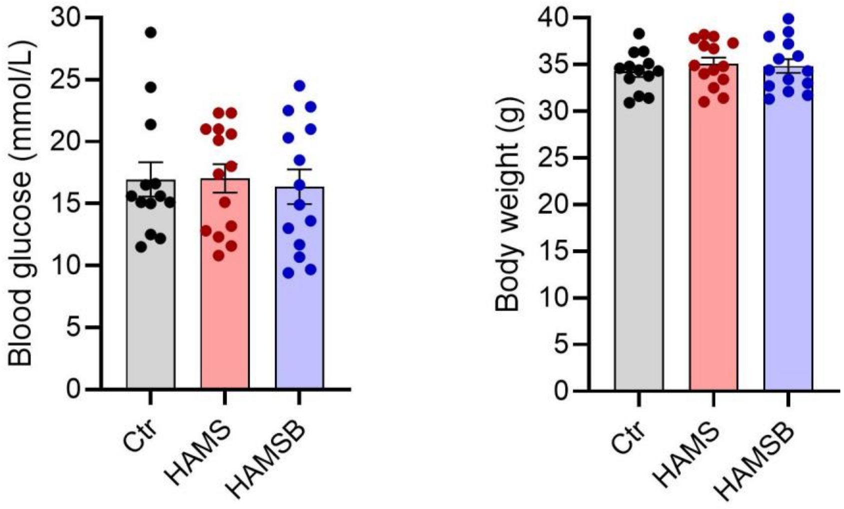

Figure S1. Baseline characteristics of db/db mice. Blood glucose (fed) and body weight were measured before initiation of the diet intervention. Bars show mean ± SEM for the three different diet groups: Ctr, HAMS and HAMSB. Ctr, Control; HAMS, high-amylose maize starch; HAMSB, butyrylated HAMS

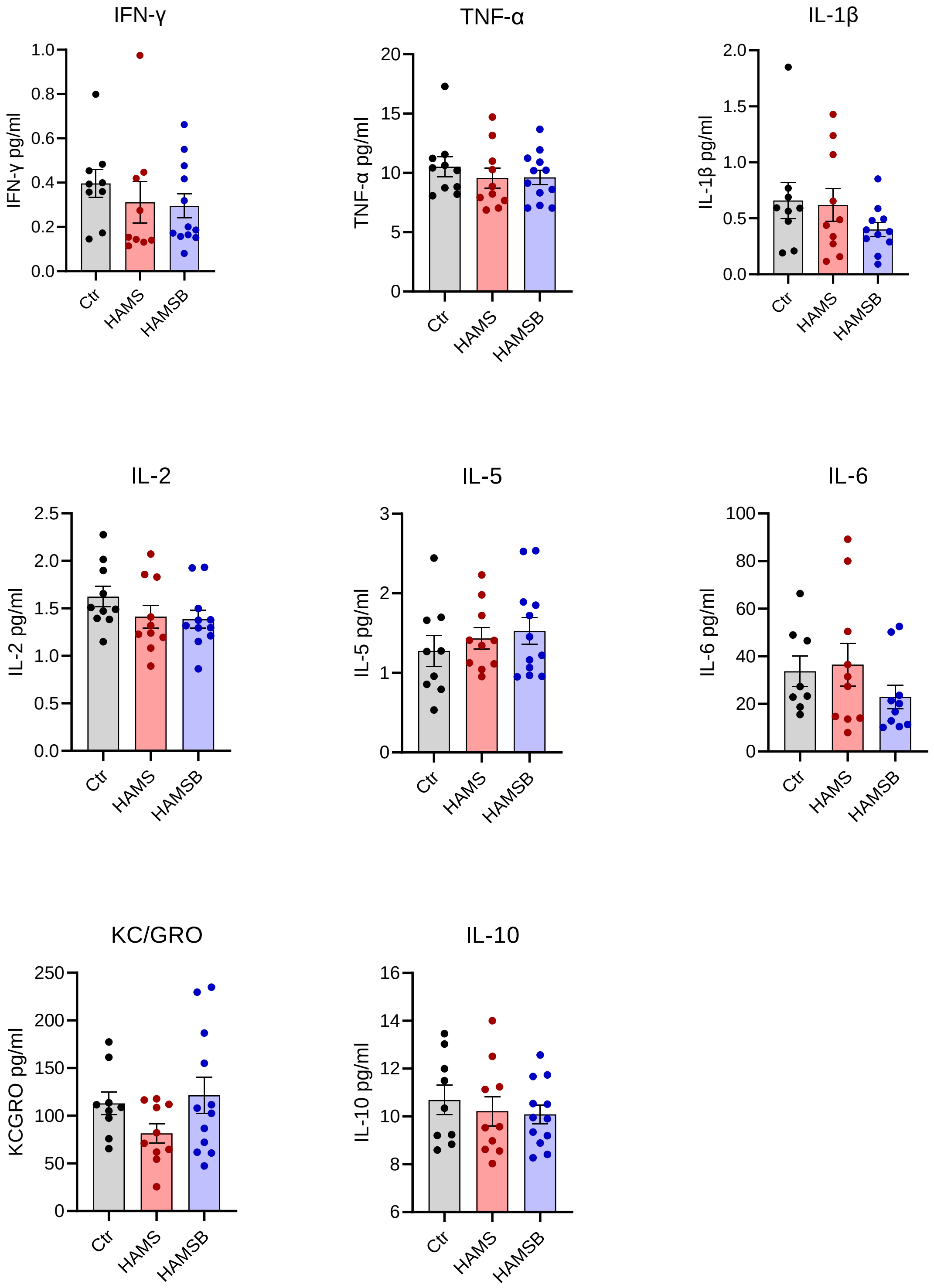

Figure S2. Cytokines in plasma. Db/db mice were fed three different diets: Ctr, HAMS or HAMSB. After 5 weeks on diet, plasma was collected for analysis of interferon gamma (IFN-γ), tumor necrosis factor alpha (TNF-α), interleukin-1 beta (IL-1β), interleukin-2 (IL-2), interleukin-5 (IL-5), interleukin-6 (IL-6), keratinocyte chemoattractant/human growth-regulated oncogene (KC/GRO) and interleukin-10 (IL-10). Bars show mean ± SEM.

Table S1. Diet formulations.

Numbers indicate g/kg

	Ctr	HAMS	HAMSB
**HAMS**	-	150	-
**HAMSB**	-	-	150
**Casein**	200	200	200
**Sucrose**	100	100	100
**Corn starch**	397.5	247.5	247.5
**Maltodextrin**	132	132	132
**Soybean oil**	70	70	70
**Cellulose**	50	50	50
**Mineral Mix, AIN-93G-MX (94046)**	35	35	35
**Vitamin Mix, AIN-93-VX (94047)**	10	10	10
**L-cystine**	3	3	3
**Choline Bitartrate**	2.5	2.5	2.5

	% by weight	% kcal from
**Protein**	17.7	18.8
**Carbohydrate**	60.1	63.9
**Fat**	7.2	17.2
**kcal/g**	3.8	

Table S2. Primers used for RT-qPCR on liver and fat tissue.

	Forward primer	Reverse primer
Fasn	5’ TCCAGTTAGAGCAGGACAAGC 3’	5’ GGAGGAGACCCAGCGGAT 3’
Scd1	5’ ACATGCTGTTTTGATGCTAGGG 3’	5’ GAAGAAACAAAGCTGGACACCA 3’
Srebp1c	5’ AGACCCTGGTGAGTGGAGG 3’	5’ TGGATGGGCAGTTTGTCTGT 3’
Acaca	5’ AGGCACTCTGATCTGGTCAC 3’	5’ ATAGCTTCCAGGAGCAGTCG 3’
Cd36	5’ GATGTGCAAAACCCAGATGA 3’	5’ GATGTGCAAAACCCAGATGA 3’
Cpt1a	5’ GGCATAAACGCAGAGCATTCCTG 3’	5’ CAGTGTCCATCCTCTGAGTAGC 3’
Pck1	5’ ATGAAAGGCCGCACCATGTA 3’	5’ GCACAGATATGCCCATCCGA 3’
G6pase	5’ TGGTAGCCCTGTCTTTCTTTG 3’	5’ TTCCAGCATTCACACTTTCCT 3’
Cd68	5’ ATGCCACAGTTTCTCCCACC 3’	5’ ACGTGTAGTTCCCAAGAGCC 3’
F4/80	5’ ACTTTCAAGGCCCAGGAGTG 3’	5’ TGCAGACTGAGTTAGGACCAC 3’
TNF-a	5’ TGTACCTTGTCTACTCCCAGGT 3’	5’ GCAGAGAGGAGGTTGACTTTC 3’
Ppia	5’ GAGCTGTTTGCAGACAAAGTTC 3’	5’ CCCTGGCACATGAATCCTGG 3’

Table S3. Primers used for RT-qPCR on islets.

Ppia	Rn00690933_m1
Ucn3	Mm00453206_s1
Pdx1	Mm00435565_m1
MafA	Rn00845206_s1
Ins1	Mm01950294_s1
Ins2	Mm00731595_gh
